# Dataset on the toxic effects of aflatoxin and ochratoxin a on the human gastric smooth muscle cells

**DOI:** 10.1016/j.dib.2019.104089

**Published:** 2019-06-04

**Authors:** Oluwadara Pelumi Omotayo, Abiodun Olusola Omotayo, Olubukola Oluranti Babalola, Mulunda Mwanza

**Affiliations:** Food Security Niche Area, North West University, South Africa

**Keywords:** Aflatoxin, Elisa, Health, Human, Gastric smooth muscle cell

## Abstract

This dataset determined the effects of aflatoxins (B_1_, B_2_, G_1_ and G_2_) and Ochratoxin A extracted from ginger collected purposively from different retails in Mafikeng, North West province of South Africa, on the Human Gastric Smooth Muscle Cells. Hundred samples of ginger were collected and utilized for this purpose and the above mentioned toxins were extracted from the ginger, screened for on ELISA, quantified by HPLC and were exposed to the cells both individually and in combination (i.e aflatoxin and ochratoxin were combined) at different concentrations (high, moderate and low) using the human interferon beta kit. They were incubated for 12 h after which the level/concentration of human interferon produced was analyzed using the ELISA.

**Table:** Specifications Table

Subject area	*Biology*
More specific subject area	*Cell Biology*
Type of data	*Table, graph, figure*
How data was acquired	*HPLC Quantification* *ELISA Screening* *Analysis on Microsoft Excel 2010*
Data format	*Raw and Analyzed*
Experimental factors	*Ginger samples collected were chopped and stored at* stored at 4 °C in a fridge prior to extraction and analysis to prevent the growth of mould and to avoid putrefaction of the samples. All the samples were then extracted and analyzed according to Ref. [4], method. Healthy human gastric smooth muscle cells were procured from Science cell, South Africa, transported on dry ice to North-West University, Mafikeng campus and stored in liquid nitrogen (−196 °C) prior to use
Experimental features	*Aflatoxins B*_*1*_*, B*_*2*_*, G*_*1*_*and G*_*2*_*were extracted from the ginger samples and exposed to the human gastric smooth muscle cell to determine the effect of the mycotoxins on the cell using the human* interferon beta ELISA kit
Data source location	*Mafikeng, North west province, South africa*
Data accessibility	*Data is with this article*

**Table:** 

**Value of the data** • It can help to create more awareness of the presence of aflatoxins and Ochratoxin A in spices especially ginger. • The effect of these mycotoxins on the human gastric cells and their reaction with human interferon beta as revealed in this dataset can serve as an insight for further investigations. • It reveals the role of aflatoxins and Ochratoxin A in the inhibition of the immune system (human interferon beta). • It can enhance strict control of ginger quality through workable set guidelines to control mycotoxins in the above mentioned crop in developing countries such as South Africa.

## 1 Data

**Fig. 1.0:**
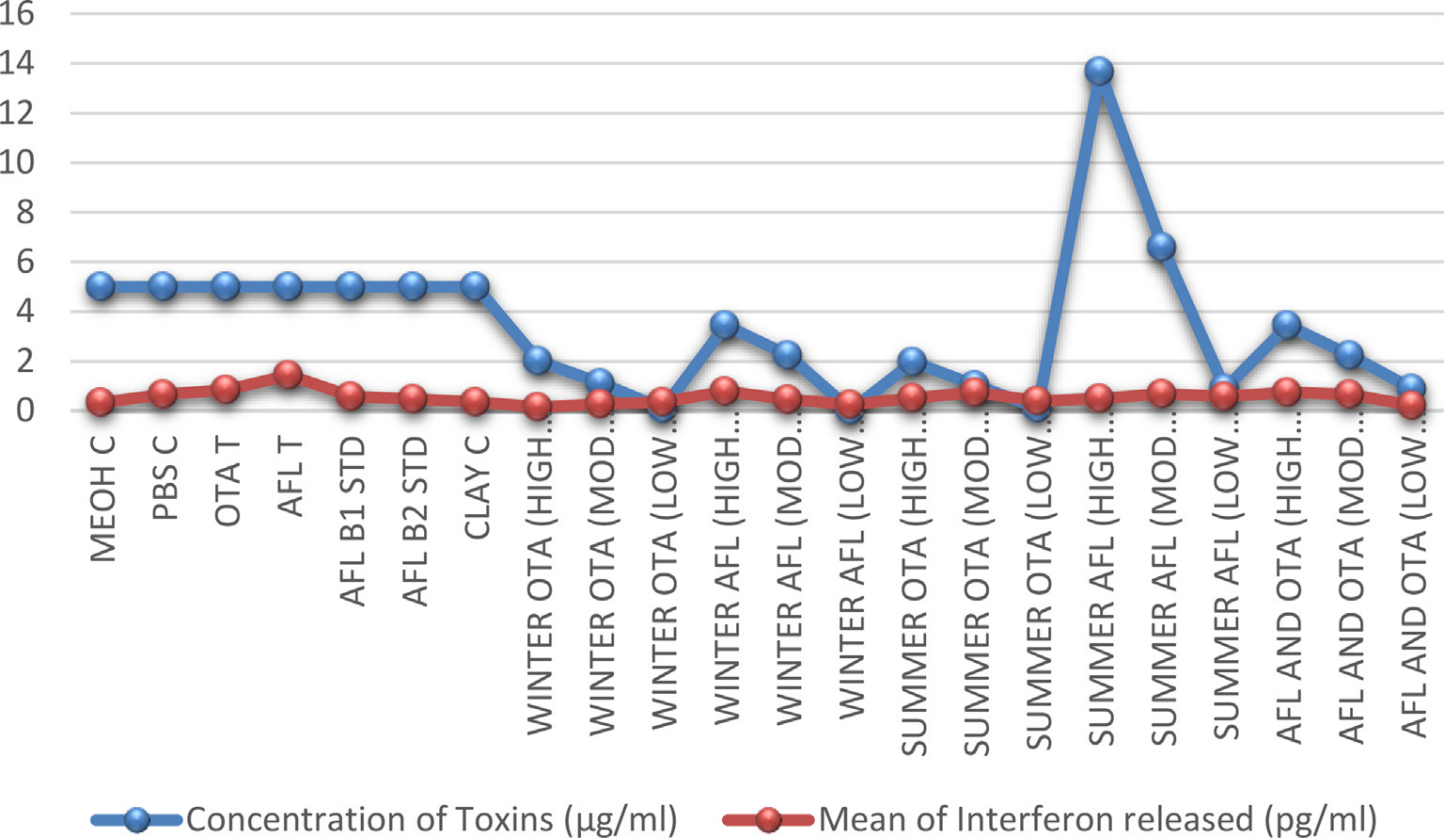
Chart of comparison of interferon expression against mycotoxin concentration. Where MEOH means methanol; PBS means Phosphate Buffer Saline; C means Control; STD means Standard; OTA means Ochratoxin; AFL means Aflatoxin.

The data in this article originated from the exposure of aflatoxins B_1_, B_2_, G_1_ and G_2_ and Ochratoxin A extracted from ginger obtained from North West province of South Africa, Mafikeng to be precise on human gastric smooth muscle cell using the human interferon beta kit and analyzed using ELISA. Table 1.0 shows the concentrations of mycotoxins used (singularly and in combination), the concentration of interferon produced and their mean value. Fig. 1.0 supports Table 1.0 by comparing the concentration of mycotoxins with the mean concentration of interferon released.

**Table 1.0:** Toxicity test conducted on cell.

Extracts	Concentration (μg/ml)	Range (interferon Released) (pg/ml)	Mean (pg/ml)
MeoH Control	5	0.32–0.36	0.34
PBS Control	5	0.53–0.79	0.66
Ochratoxin A total	5	0.50–0.91	0.84
Aflatoxin total	5	1.38–1.47	1.43
Aflatoxin B1 Standard	5	0.32–0.62	0.57
Aflatoxin B2 Standard	5	0.28–0.66	0.48
Clay Control	5	0.02–1.07	0.36
Winter OTA (High conc)	2.014	0.05–0.27	0.15
Moderate conc.	1.107	0.19–0.44	0.27
Low conc.	0.096	0.21–0.47	0.36
Winter Afl (High conc)	3.44	0.67–0.92	0.78
Moderate conc.	2.24	0.00–0.84	0.46
Low conc.	0.02	0.01–0.64	0.25
Summer OTA (High conc)	1.969	0.34–1.28	0.52
Moderate conc.	1.028	0.05–0.58	0.74
Low conc	0.097	0.03–0.12	0.40
Summer Aflatoxin (High conc)	13.67	0.23–0.42	0.50
Moderate conc	6.59	0.32–1.14	0.69
Low conc	0.89	0.46–1.02	0.58
Aflatoxin and OTA(high conc)	3.44	0.20–0.74	0.74
Aflatoxin and OTA(Moderate conc)	2.24	0.06–0.24	0.65
Aflatoxin and OTA(low conc)	0.89	0.01–0.16	0.20
Total		6.18–15.16	11.27

## 2 Sample preparation

Ginger samples were chopped into pieces and covered in sample bottles and stored at 4 °C in a fridge prior to extraction and analysis to prevent the growth of mould and to avoid putrefaction of the samples Omotayo et al. [1], [2]. All the samples were then extracted and analyzed according to Ref. [4], method. Healthy human gastric smooth muscle cells were procured from Science cell, South Africa, transported on dry ice to North-West University, Mafikeng campus and stored in liquid nitrogen (−196 °C) prior to use.

## 3 Materials

These include Beaker, Flat bottom flask, Whatmann filter paper, Shaker, vortex, Phosphate buffer salt, Methanol, Ethanol, Sodium Chloride, Immunoaffinity column and Aflatoxin kits.

## 4 Mycotoxin extraction

Aflatoxin and ochratoxin extraction were done according to Refs. [3], [4] method respectively with slight modifications in the quantity of samples used for ochratoxin extraction (10g of ginger sample was used instead of 25g used by Maryam and Jinap)

## 5 Toxicological analysis

Human Gastric Smooth Muscle Cell with catalogue number #2810 was obtained from Science Cell Research Laboratories. It was cultured in line with the instructions on the user's guide).

Harvested cells were seeded into a 96 well plate at approximately 5000 cells per well. Methanol, phosphate buffer saline, Ochratoxin A and aflatoxin standards and clay that were used for the control test were respectively added into five wells containing the cells. After which the aflatoxins extracts from ginger were added to the cells, in the order of high, moderate and low concentration (concentrations are presented in Table 1.0).

Ochratoxin A extracted from ginger was also added to the wells in the same order as aflatoxins. The ginger extracts which contain both aflatoxins and Ochratoxin A were also added into separate wells in the order stated above. Negative samples with no mycotoxin were also added into separate wells (all the concentrations were tested in one batch) and thereafter placed in a 5% CO2 incubator overnight for twelve (12) hours. After twelve hours the cells were transferred to the human interferon beta ELISA kit (E-EL-H0085) and the assay was carried out according to the kit's user's guide as follows:

About 100μl of the standard obtained from the kit was added to each well, the concentrations of the standard used are (2000, 1000, 500, 250, 125, 61.5, 31.25 pg/mL) picogram, blank well was added with reference standard and sample diluent and incubate for 90 min at 37°c, the liquid was removed and 100μl of biotinylated detection ab was added and incubated for one (1) hour at 37°c, after which it was aspirated and the kit was washed three (3) times, 100μl of HRP Conjugate was added and incubated for thirty (30) minutes at 37 °C, it was aspirated and the kit was washed five (5) times, 90 μl of substrate reagent was added and incubated for fifteen (15) minutes at the same temperature, 50 μl of stop solution was added and the data were read on a microplate reader at 450nm immediately.

This test utilized the extracted mycotoxins from the ginger in order to establish the effect of these mycotoxins present in ginger.

## 6 Statistical analysis

Data generated was analyzed using Microsoft Excel- 2010 to calculate mean, standard deviations and ranges of the different mycotoxins and statistical differences between seasons where applicable.

## 7 Mycotoxins toxicity determination using the human interferon β kit

The determination of mycotoxin extracts toxicity was done using the human interferon beta kit. To evaluate the dose response of standards, extracts of ginger, Aflatoxins (AFs), Ochratoxin A (OTA), and combination of Aflatoxins and Ochratoxin A on the human gastric cell. An increase of interferon beta was recorded with an increase of aflatoxin and Ochratoxin A concentration, the data obtained are summarised in Table 1.0 and Fig. 1.0 below.
